# Hydrogen sulfide protects against toxicant acrolein-induced ferroptotic cell death in Sertoli cells

**DOI:** 10.3389/fphar.2024.1440147

**Published:** 2024-08-01

**Authors:** Zhimin Mao, Qun Ji, Ping Chen, Kun Zhong, Xuhui Zeng

**Affiliations:** Institute of Reproductive Medicine, Medical School, Nantong University, Nantong, Jiangsu, China

**Keywords:** acrolein, male infertility, Sertoli cell, ferroptosis, hydrogen sulfide, oxidative stress

## Abstract

Acrolein (ACR) is a ubiquitous environmental pollutant and byproduct of lipid peroxidation that has been implicated in male infertility. However, the molecular mechanisms underlying ACR-induced toxicity in Sertoli cells remain unclear. Given its role in inducing oxidative stress, we examined whether ferroptosis, an iron-dependent form of regulated cell death, could mediate ACR toxicity in Sertoli cells. We also tested if hydrogen sulfide (H_2_S), which has antioxidant and ACR detoxifying properties, could protect Sertoli cells from ACR-induced ferroptosis. ACR exposure decreased Sertoli cell viability, increased protein carbonylation and p38 MAPK phosphorylation, indicating oxidative injury. ACR also depleted glutathione (GSH), downregulated the cystine importer SLC7A11, increased intracellular ferrous iron (Fe^2+^) and lipid peroxidation, suggesting activation of ferroptosis. Consistently, the ferroptosis inhibitor deferoxamine (DFO) markedly attenuates ACR-induced cell death. Further studies revealed that ACR-induced ferroptotic changes were prevented by exogenous H_2_S and exaggerated by inhibition of endogenous H_2_S production. Furthermore, H_2_S also suppressed GPX4 inhibitor RSL3-induced intracellular ACR accumulation and ferroptosis. In summary, our study demonstrates that ACR induces ferroptotic cell death in Sertoli cells, which can be prevented by H_2_S through multiple mechanisms. Targeting the H_2_S pathway may represent a therapeutic strategy to mitigate ACR-induced Sertoli cell injury and preserve male fertility.

## 1 Introduction

Infertility affects more than 15% of couples of reproductive age worldwide, posing a serious global health problem. Male factor infertility, often due to declines in sperm quantity and quality, accounts for up to 50% of infertile couples (Agarwal et al., 2021). Environmental toxicants and unhealthy lifestyles are major contributors to impaired sperm production and function (Ilacqua et al., 2018; Rodprasert et al., 2023). Therefore, understanding the effects and mechanisms of these factors on testicular function, particularly spermatogenesis, is imperative to develop preventive strategies against male infertility.

Sertoli cells are the somatic cells of the testes that support spermatogenesis by providing structural and nutritional support to developing germ cells. As a component of the blood-testis barrier (BTB), Sertoli cells also protect germ cells from toxins and other insults (Ghafouri-Fard et al., 2021; Thumfart and Mansuy, 2023). Because of this reason, Sertoli cells themselves are vulnerable to damage from toxicants like cadmium, arsenic, bisphenol A and reactive aldehydes, resulting in impaired spermatogenesis and infertility (Liu et al., 2011; Zhang et al., 2017; Ramos-Treviño et al., 2018; Mao et al., 2023a). Elucidating the mechanisms of Sertoli cell injury and identifying protective agents are thus important areas of research.

Acrolein (ACR) is a ubiquitous α,β-unsaturated aldehyde found in the environment, diet, and endogenously produced through lipid peroxidation and other metabolic processes (Stevens and Maier, 2008). ACR exposure is associated with smoking, alcohol consumption, pollution, diabetes, and cardiovascular diseases—all risk factors for male infertility (Mahalingam et al., 2017; Sansone et al., 2018; Del Giudice et al., 2020; Huang et al., 2024). Importantly, ACR levels are exceptionally high in the male reproductive system as sperm membranes are rich in polyunsaturated fatty acids (PUFAs), which produce high levels of endogenous ACR during lipid peroxidation (Gautier and Aurich, 2022). Consistently, high ACR levels have been detected in mature sperm (Moazamian et al., 2015). The presence of ACR in the male reproductive system could cause toxic effects and lead to infertility. The cytotoxicity of ACR has been shown to be stems mainly from its reactivity with proteins, lipids, and DNA. ACR readily forms adducts with these biomolecules, disrupting their structure and function. Additionally, ACR induces oxidative stress by depleting glutathione (GSH) and inhibiting antioxidant enzymes (Moghe et al., 2015). We and others have shown ACR causes oxidative damage in testicular Sertoli cells, which can lead to the impairment of spermatogenesis (Liu et al., 2011; Mao et al., 2023a). Despite the established link between ACR and impaired male reproductive function, the precise molecular mechanisms underlying its detrimental effects on Sertoli cells and spermatogenesis remain elusive. Further research is warranted to unravel these mechanisms and identify potential therapeutic interventions to mitigate ACR-induced reproductive toxicity.

As of the downstream cellular event, ferroptosis has been drawn great attention. Ferroptosis is an iron-dependent form of programmed cell death caused by a redox imbalance between the production of oxidants and antioxidants. Ferroptosis is characterized by depletion of GSH, inactivation of the enzyme glutathione peroxidase 4 (GPX4), and unchecked lipid peroxidation. This results in the accumulation of toxic lipid peroxidation products such as ACR and 4-hydroxynonenal (4-HNE) (Wang et al., 2023). As a reactive species, ACR is implicated not only in the induction of oxidative stress, but also as a driving factor for oxidative stress and an effector molecule for ferroptosis. It is therefore conceivable that ACR could be critically involved in Sertoli cell injury, potentially through induction of oxidative stress and ferroptosis.

Hydrogen sulfide (H_2_S) is an endogenous gasotransmitter with antioxidant and cytoprotective effects in many tissues. It is generated enzymatically in cells by cystathionine β-synthase (CBS), cystathionine γ-lyase (CSE), and 3-mercaptopyruvate sulfurtransferase (3-MST) (Kimura, 2010). All three H_2_S-producing enzymes are expressed in the testes, implying a physiological role in the male reproductive system (Sugiura et al., 2005; Li et al., 2015). Emerging evidence indicates H_2_S counters oxidative stress in various cell models through multiple mechanisms (Wu et al., 2015; Mao et al., 2019; Hu et al., 2020). The multi-faceted effects of H_2_S on oxidative stress make it an intriguing potential therapy for ACR-induced toxicity. In this study, we speculated that the toxicity of ACR on Sertoli cells could be related to ferroptosis, which could be prevented by H_2_S. The aim of this study was to test this hypothesis.

Here, we present our results that ACR caused Sertoli cell injury by inducing ferroptosis, and H_2_S protected against Sertoli cell ferroptosis induced by ACR and other stimuli. H_2_S could be developed to prevent and treat ferroptotic Sertoli cell injury.

## 2 Materials and methods

### 2.1 Materials

Acrolein (ACR, #ZD816) was obtained from Xianding Biotechnology Co., Ltd. (Shanghai, China). Sodium hydrosulfide hydrate (NaHS, #161527) and deferoxamine (DFO, #D9533) were purchased from Sigma-Aldrich (St Louis, MO, United States). β-cyano-L-Alanine (BCA, #10010947) was bought from Cayman Chemical (Ann Arbor, MI, United States). RSL3 (#HY-100218A) was from MedChemExpress (MCE, Shanghai, China). Anti-rabbit antibody against SLC7A11 (#26864-1-AP) was from Proteintech (Chicago, IL, United States). Anti-rabbit antibody against phospho-p38MAPK (Thr180/Tyr182) (#8203S) and anti-mouse antibody against α-tubulin (#3873T) were from Cell Signaling Technology (Danvers, MA, United States). Anti-mouse β-actin (#GB12001) antibody was from Servicebio Technology (Wuhan, China). Anti-rabbit IgG (#5366), and anti-mouse IgG (#5470) were from Cell Signaling Technology (Danvers, MA, United States).

### 2.2 Cell culture

Mouse Sertoli TM4 cells (#CRL-1715) were obtained from the American Type Culture Collection (ATCC, Rockville, MD, United States). Cell culture was performed as we previously reported (Mao et al., 2023a). For routine maintenance, the cells were cultured in Dulbecco’s Modified Eagle Medium (DMEM)/F-12 (#11320-033, Invitrogen) supplemented with 10% fetal bovine serum (FBS, #F8687, Sigma-Aldrich). For experiments, the cells were cultured in DMEM/F-12 containing 1% FBS.

### 2.3 Assessment of cell viability

To evaluate cell viability, a double staining protocol with Calcein-AM and propidium iodide (PI) was utilized (#C542, Dojindo). Briefly, cells in 96-well plates were incubated with a mixture of Calcein-AM and PI for 10–20 min. Viable cells were stained green with Calcein-AM due to intracellular esterase activity, while dead cells with compromised plasma membranes were stained red with PI. Stained cells were visualized and imaged under a fluorescence microscope.

Alternatively, cell viability was quantified using the WST reagent (#CK04, Dojindo). Cells cultured in 96-well plates were incubated with WST for approximately 30 min, and the absorbance at 450 nm was measured using a spectrometer.

### 2.4 Western blot analysis

Western blot was performed as described previously (Mao et al., 2023a). Briefly, equal amounts of protein samples were separated by SDS-PAGE gels and transferred to PVDF membranes (#IPVH00010, Merck Millipore). The membranes were blocked with 3% bovine serum albumin (BSA) in phosphate-buffered saline (PBS) and incubated with primary antibodies followed by secondary antibodies. Protein bands were detected using Amersham Imager 600 (GE Healthcare). To confirm equal protein loading, the membranes were reprobed with anti-α-tubulin or β-actin antibody. Densitometry analysis was performed using ImageJ software and normalized to the respective control.

### 2.5 Assessment of protein carbonylation with oxyblot

Protein carbonylation was examined using the OxyBlot Protein Oxidation Detection Kit (#S7150, EMD Millipore) as described previously (Mao et al., 2023a). Briefly, 5–10 μg of protein samples were denatured with 12% SDS and derivatized with 2,4-dinitrophenylhydrazine (DNPH). The DNP-derivatized protein samples were processed for standard Western blotting. To confirm equal protein loading, the blots were stained with EZBlue Gel Staining Reagent (#G1041-500ML, Sigma-Aldrich).

### 2.6 Quantification of GSH

Intracellular GSH levels were measured using a GSSG/GSH quantification kit from Dojindo (#G263). Briefly, the treated cells were lysed with 80 µL 10 mM HCl, followed by addition of 20 µL of 5% 5-sulfosalicylic acid. After collecting the supernatant by centrifuging the lysate at 4°C, GSH content in the supernatants was quantified by measuring absorbance at 405 nm using a microplate reader, according to the manufacturer’s protocol.

### 2.7 Detection of intracellular ferrous iron (Fe^2+^)

Intracellular Fe^2+^ was detected using the FerroOrange fluorescent probe from Dojindo (#F374). After treatments, cells were washed and incubated with 1 μM FerroOrange solution for 30 min at 37°C. Fluorescence microscopy images were acquired and analyzed using ImageJ software. The mean fluorescence intensity of each treatment group was normalized to that of the control.

### 2.8 Assessment of lipid peroxidation

Lipid peroxidation was assessed utilizing the fluorescent probe C11 BODIPY 581/591 (#GC40165, GlpBio). Briefly, after treatments, cells were incubated with 2.5 μM C11 BODIPY 581/591 dye in fresh culture medium for 30 min at 37°C. Subsequently, the cells were washed, imaged under a fluorescence microscope, and fluorescence intensity was quantified using ImageJ software. The mean fluorescence intensity of each treatment group was normalized to that of the control group.

### 2.9 Measurement of intracellular ACR

Relative intracellular ACR levels were examined utilizing AcroleinRED from funakoshi (#FDV-0022). After treatments, culture medium was removed and replaced with medium containing 20 μM AcroleinRED. Following 30–60 min incubation, cells were washed, imaged under a fluorescence microscope, and fluorescence intensity was quantified using ImageJ software. The mean fluorescence intensity of each treatment group was normalized to that of the control.

### 2.10 Statistical analysis

The data are expressed as mean ± standard error (S.E.). Statistical comparisons between two groups were performed using Student’s t-test. For comparisons of multiple groups, one-way analysis of variance (ANOVA) followed by Dunnett’s *post hoc* test was utilized. Statistical analyses were conducted using either Microsoft Excel (Microsoft Corporation, Redmond, WA, United States) or Sigmaplot software (Systat Software Inc., San Jose, CA, United States). In all cases, a *P*-value less than 0.05 was considered statistically significant.

## 3 Results

### 3.1 ACR induces hallmarks of ferroptosis in Sertoli cells

As we have reported that ACR induces cell death in cultured Sertoli cells (Mao et al., 2023a). Here, we repeated some of the experiments and found that ACR caused concentration-dependent cell death (Supplementary Figures S1A, B). This effect of ACR was associated with an early elevation in protein carbonylation and activation of the oxidative stress-sensitive P38, suggesting induction of oxidative cell death (Supplementary Figure S1C).

**FIGURE 1:**
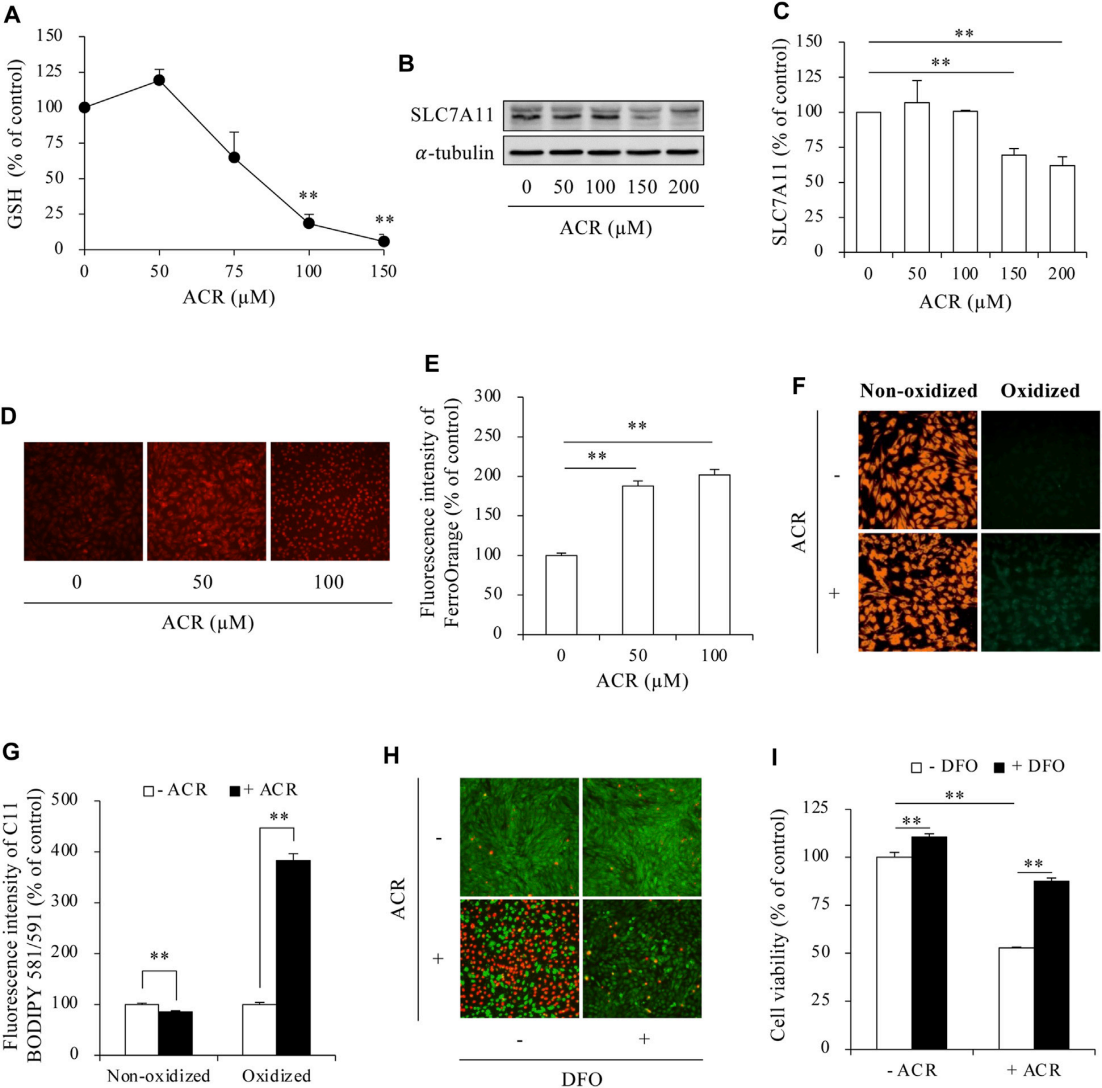
ACR induced ferroptosis in Sertoli cells. **(A)** Effect of ACR on intracellular GSH level. TM4 cells were cultured with the indicated concentrations of ACR for 3 h. The cellular lysates were analyzed for GSH levels with GSSG/GSH quantification kit. Data are mean ± S.E. (n = 3; **P < 0.01 vs. control). **(B,C)** Effect of ACR on the protein level of SLC7A11. TM4 cells were incubated with the indicated concentrations of ACR for 3 h, and the cellular lysates were used for protein expression of SLC7A11 **(B)**. Densitometric quantitation of these bands was shown in **(C)**. Data in **(C)** are mean ± S.E. (n = 3; **P < 0.01). **(D,E)** Effect of ACR on intracellular Fe^2+^ level. TM4 cells were stimulated with the indicated concentrations of ACR for 3 h. FerroOrange staining was conducted for Fe^2+^ detection. **(F,G)** Induction of lipid peroxidation by ACR. TM4 cells were cultured with 100 µM ACR for 3 h, followed by staining with C11 BODIPY 581/591 for detection of lipid oxidation. Densitometric quantitation of relative fluorescence intensity in **(D,F)** was performed on 20 cells randomly selected from each group, and the data were shown in **(E,G)**, respectively. (n = 20; **P < 0.01). **(H,I)** Effect of DFO on ACR-induced Sertoli cell death. TM4 cells were pretreated with or without 400 µM DFO for 30 min before exposing to 100 µM ACR for an additional 6 h. The cell viability was determined by Calcein-AM/PI staining **(H)** and WST assay **(I)**. Data in **(I)** are mean ± S.E. (n = 5; **P < 0.01).

To investigate whether ferroptosis underlies ACR cytotoxicity, we analyzed several ferroptosis markers. ACR exposure caused a concentration-dependent reduction in intracellular GSH, which was associated with a downregulation of the cystine transporter SLC7A11 (Figures 1A–C). Further Investigation demonstrated that ACR exposure led to an increase in intracellular Fe^2+^ levels. This was evidenced by the heightened fluorescence intensity observed within the cells following the addition of FerroOrange, a fluorescent probe specifically designed to detect Fe^2+^ (Figures 1D, E). Additionally, ACR also caused lipid peroxidation (Figures 1F, G) as detected by using a fluorescent dye C11 BODIPY 581/591. This dye emits red fluorescence when bound to non-oxidized lipids. However, when lipids become oxidized, the dye shifts its fluorescence to the green range. ACR exposure decreased the red fluorescence, while it increased green fluorescence, indicating an induction of lipid oxidation. In further support of an involvement of ferroptosis in ACR-induced cell death, the ferroptosis inhibitor deferoxamine (DFO, an iron chelator that prevents iron-dependent lipid peroxidation) significantly prevented ACR-induced cell death, as revealed by assessment of cell viability using Calcein-AM/PI staining and WST assay (Figures 1H, I). Collectively, these data show that ACR triggered ferroptotic cell death in Sertoli cells.

### 3.2 H_2_S inhibits ACR-induced ferroptosis in Sertoli cells

**FIGURE 2:**
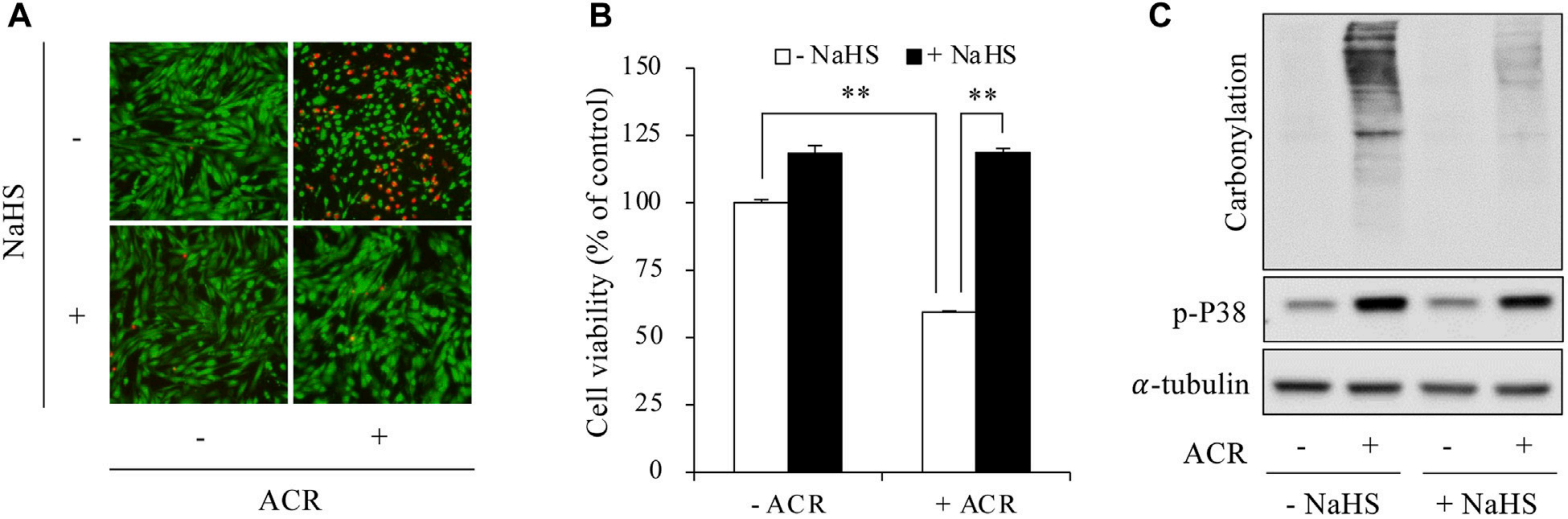
H_2_S prevented ACR-induced oxidative injury in Sertoli cells. **(A,B)** Amelioration of Sertoli cell viability by sodium hydrosulfide (NaHS). TM4 cells were pretreated with or without 1 mM NaHS for 15 min before exposing to 100 µM ACR for an additional 6 h. The cell viability was determined by Calcein-AM/PI staining **(A)** and WST assay **(B)**. Data in **(B)** are mean ± S.E. (n = 5; **P < 0.01). **(C)** Inhibition of protein carbonylation and P38 activation by NaHS. TM4 cells were exposed to 50 µM ACR with or without 1 mM NaHS for 6 h. Cellular lysates were used to analysis of protein carbonylation and P38 phosphorylation.

We have reported that H_2_S protects against ACR-induced oxidative injury in Sertoli cells (Mao et al., 2023a). Here, we tested whether the effect of H_2_S could be through suppressing ferroptosis. First, we confirmed that H_2_S donor NaHS prevented ACR-induced oxidative cell death, as evidenced by the obviously reduced number of PI-positive red cells, as well as the increased formazan formation (Figures 2A, B). This protective effects of H_2_S donor were associated with a reduction in the level of protein carbonylation and P38 phosphorylation (Figure 2C).

**FIGURE 3:**
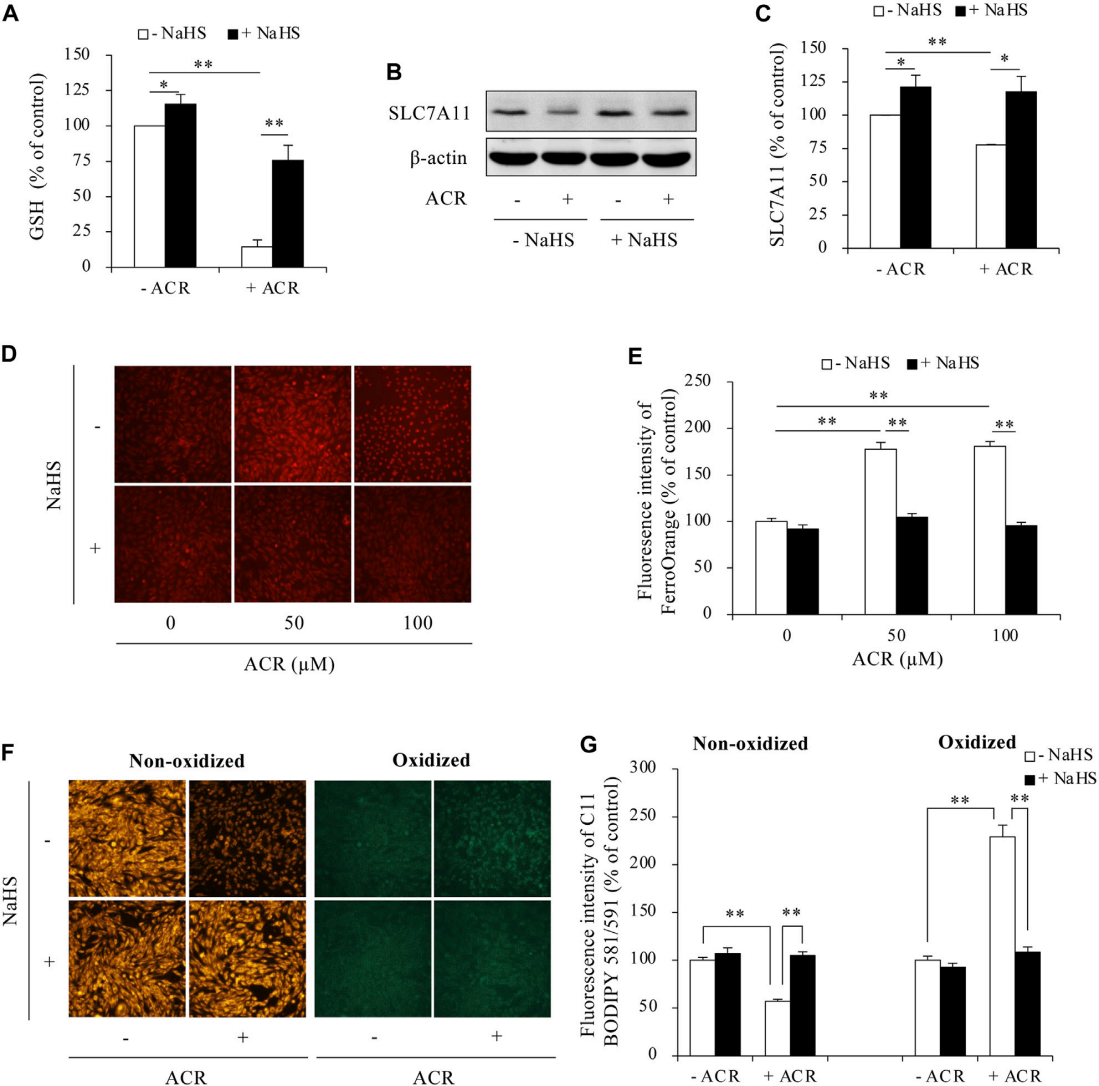
H_2_S inhibited ACR-induced ferroptosis in Sertoli cells. **(A)** Reversion of GSH level by NaHS. TM4 cells were exposed to 100 µM ACR with or without 1 mM NaHS for 3 h. The cellular lysates were analyzed for GSH levels with GSSG/GSH quantification kit. Data are mean ± S.E. (n = 4; *P < 0.05, **P < 0.01). **(B,C)** Upregulation of SLC7A11 by NaHS. TM4 cells were exposed to 100 µM ACR with or without 1 mM NaHS for 3 h. Cellular lysates were used to analysis of protein expression of SLC7A11 **(B)**. Densitometric quantitation of these bands was shown in **(C)**. Data in **(C)** are mean ± S.E. (n = 3; *P < 0.05, **P < 0.01). **(D,E)** Clearance of Fe^2+^ accumulation by NaHS. TM4 cells were stimulated with the indicated concentrations of ACR with or without NaHS for 3 h. FerroOrange staining was performed for Fe^2+^ detection. **(F,G)** Prevention of lipid peroxidation by NaHS. TM4 cells were exposed to 100 µM ACR with or without NaHS for 3 h. After that, C11 BODIPY 581/591 staining were used for indication of lipid oxidation. Densitometric quantitation of relative fluorescence intensity in **(D,F)** was performed on 20 cells randomly selected from each group, and the data were shown in **(E,G)**, respectively (n = 20; **P < 0.01).

**FIGURE 4:**
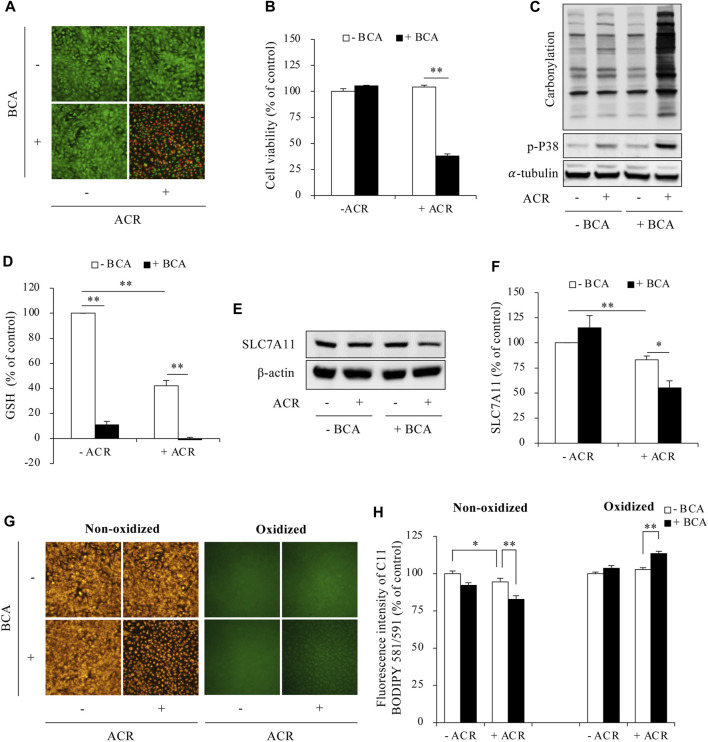
Inhibition of H_2_S promoted ferroptosis in ACR-treated Sertoli cells. **(A,B)** Acceleration of Sertoli cell death by BCA. TM4 cells were pretreated with or without 2 mM BCA for 12 h before exposing to 50 µM ACR for an additional 5 h. The cell viability was determined by Calcein-AM/PI staining **(A)** and WST assay **(B)**. Data in B are mean ± S.E. (n = 5; **P < 0.01). **(C)** Aggravation of protein carbonylation and P38 activation by BCA. TM4 cells were exposed to 50 µM ACR with or without 2 mM BCA for 2 h. Cellular lysates were subjected to analysis of protein carbonylation and P38 phosphorylation. **(D)** Acceleration of GSH consumption by BCA. TM4 cells were pretreated with or without 2 mM BCA for 6 h before exposing to 50 µM ACR for an additional 3 h. The cellular lysates were analyzed for GSH levels with GSSG/GSH quantification kit. Data are mean ± S.E. (n = 4; **P < 0.01). **(E,F)** Aggravation of downregulated protein expression of SLC7A11 by BCA. TM4 cells were incubated with 75 µM ACR for 3 h, and the cellular lysates were used for protein expression of SLC7A11 **(E)**. Densitometric quantitation of these bands was shown in **(F)**. Data in **(F)** are mean ± S.E. (n = 3; *P < 0.05, **P < 0.01). **(G,H)** Promotion of lipid peroxidation by BCA. TM4 cells were exposed to 50 µM ACR with or without 2 mM BCA pretreatment for 3 h. After that, C11 BODIPY 581/591 staining were used for analyzing lipid oxidation. Densitometric quantitation of relative fluorescence intensity in **(G)** was calculated on 20 cells randomly selected from each group, and the data were shown in **(H)** (n = 20; *P < 0.05, **P < 0.01).

Then, we determined the possible involvement of ferroptosis. As shown in Figure 3, supplementing cells with exogenous H_2_S elevated intracellular GSH levels, upregulated SLC7A11 expression, and blocked Fe^2+^ and lipid peroxidation accumulation. Consistently, inhibiting endogenous H_2_S synthesis with BCA exerted the opposite effects. It exacerbated ACR toxicity (Figures 4A, B) and ACR-induced cellular oxidation (Figure 4C) as we have previously documented (Mao et al., 2023a). It also significantly potentiated ACR-induced changes in ferroptosis markers, including markedly reduced GSH/SLC7A11 level (Figures 4D–F) and elevated lipid peroxidation (Figures 4G, H). Together, these findings indicate H_2_S protects Sertoli cells from ACR by inhibiting ferroptosis.

### 3.3 H_2_S inhibits RSL3-induced ferroptosis in Sertoli cells

**FIGURE 5:**
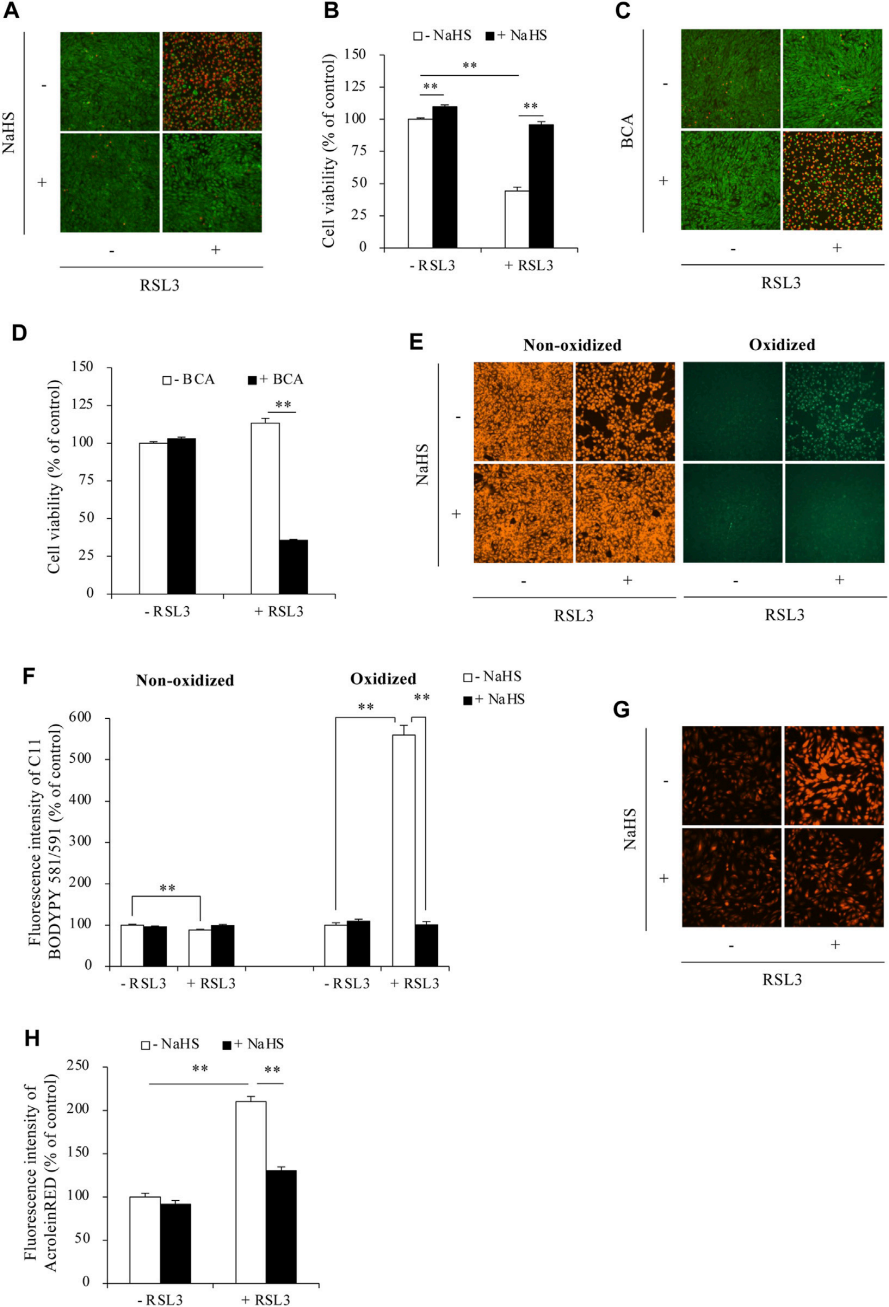
H_2_S protected against RSL3-induced ferroptosis in Sertoli cells. **(A–D)** Effect of H_2_S on RSL3-induced Sertoli cell death. TM4 cells were incubated with 5 µM RSL3 with or without 1 mM NaHS for 24 h **(A)** or in the presence or absence of 2 mM BCA for 16 h **(B)**. The cell viability was determined by Calcein-AM/PI staining **(A,C)** and WST assay **(B,D)**. Data in **(B,D)** are mean ± S.E. (n = 4; **P < 0.01). **(E,F)** Effect of H_2_S on RSL3-induced lipid peroxidation. TM4 cells were exposed to 5 µM RSL3 with or without 1 mM NaHS for 16 h. After that, C11 BODIPY 581/591 staining were subjected to analysis of lipid oxidation. **(G,H)** Effect of H_2_S on RSL3-induced ACR accumulation. TM4 cells were incubated with 10 µM RSL3 with or without 1 mM NaHS for 6 h. AcroleinRED staining was performed for ACR detection. Densitometric quantitation of relative influorescence intensity in **(E,G)** was performed on 20 cells randomly selected from each group, and the data were shown in **(F,H)**, respectively (n = 20; **P < 0.01).

Given the suppressive effect of H_2_S on ACR-induced ferroptosis, we examined whether this extends to other ferroptosis triggers using the GPX4 inhibitor RSL3. Figure 5 shows that exogenous H_2_S donor NaHS also prevented RSL3-induced cell death (Figures 5A, B). On the contrary, inhibition of endogenous H_2_S with BCA exacerbated cell death (Figures 5C, D). Consistent with the protective actions of H_2_S donor on cell death, NaHS also significantly suppressed RSL3-induced changes in ferroptotic markers. It significantly reduced RSL3-triggered lipid peroxidation (Figures 5E, F). Intriguingly, as one of the lipid peroxidation products, RSL3 inhibition dramatically elevated the endogenous level of ACR. However, in the presence of H_2_S donor, this accumulation of ACR could be completely abolished (Figures 5G, H). These observations suggest that besides ACR, H_2_S could also inhibit ferroptosis induced by other stimuli in Sertoli cells.

## 4 Discussion

In this study, we demonstrated that H_2_S prevented ACR-induced ferroptosis in Sertoli cells via multiple mechanisms. Given that the ACR is a key toxic chemical underlying reproductive injury under various situations, our finding could have significant implications.

ACR is a ubiquitous toxicant implicated in reproductive damage from various pathological factors. Cigarette smoke, dietary fat peroxidation, high-temperature cooking, automotive exhaust, industrial pollution, and water disinfection byproducts could all result in an increased level of ACR *in vivo*. In addition, ACR also arises endogenously through lipid peroxidation, oxidative stress, inflammation, and polyamine metabolism. Conditions like diabetes, atherosclerosis, neurodegenerative diseases, and trauma are associated with increased ACR (Stevens and Maier, 2008; Moghe et al., 2015). The elevated ACR could readily cross the BTB and exert toxic effects in testes. The highly reactive ACR has many toxic effects, such as formation of adducts with proteins, lipids and DNA, depletion of GSH, suppression of other antioxidants, and activation of cell death signaling (Stevens and Maier, 2008; Moghe et al., 2015). These molecular events interfere the process of sperm production and impairs sperm function. Moreover, damaged cells themselves can generate additional ACR, thus propagating a vicious cycle of oxidative damage that compromises male fertility (Aitken et al., 2012; Moazamian et al., 2015). Given all the factors leading to the elevated ACR have been documented to be closely related to male infertility, our findings about the effect and mechanisms of ACR toxicity and the protective strategy in Sertoli cells should have close clinical relevance.

Our results showed that ACR induced oxidative stress and triggered ferroptosis in Sertoli cells. It depleted GSH, induced protein carbonylation, activated redox signaling. These findings align with previous studies from ours and others showing that ACR induces oxidative stress via elevating reactive oxygen species (ROS) production and suppressing antioxidant enzymes levels (Taghiabadi et al., 2012; Kang, 2013; Mao et al., 2023a). One of the key mechanisms underlying the toxicity of ACR is its ability to deplete intracellular GSH levels via formation of conjugates with GSH (Shah et al., 2014; Xiong et al., 2021). In this study, we found that ACR could also reduce the protein level of SLC7A11, the cystine/glutamate antiporter responsible for cystine uptake. The effect of ACR could be an additional mechanism contributing to the reduced intracellular GSH level.

Our study further characterized that one form of the cell death initiated by ACR in Sertoli cells is ferroptosis. Besides its reported actions on ferroptosis-related molecular events, such as GSH depletion and oxidative stress induction, ACR also increased intracellular iron accumulation and promoted lipid peroxidation in Sertoli cells. ACR exposure markedly increased redox-active Fe^2+^ levels. The mechanisms by which ACR disrupted iron homeostasis are currently unclear. Potentially, it could be through 1) upregulating iron import proteins, 2) downregulating iron storage and export proteins, and/or 3) releasing iron from biomolecules by adduct formation. We have recently reported that cyclophosphamide (CYP)-induced cystitis was associated with decreased expression of the cellular iron export protein ferroportin 1 (FPN1) in bladder tissues (Mao et al., 2023b). Given that ACR is a metabolite of CYP and mediates CYP-induced cystitis, it is conceivable that ACR may exert the similar effect to affect cellular iron export, thus resulting in iron accumulation. Further analysis of the effects of ACR on the regulators of iron import/export and iron release will provide important insights into mechanisms of ACR-mediated ferroptosis and tissue injury.

Iron acts as a crucial catalyst in the process of lipid peroxidation. Consequently, the observed increase in intracellular Fe^2+^ levels induced by ACR further exacerbated lipid peroxidation, leading to the accumulation of lipid peroxidation products, including ACR itself. This creates a detrimental cycle where ACR-initiated oxidative damage fuels further ACR generation, thereby perpetuating cellular injury and ultimately culminating in cell death. Breaking this vicious cycle is therefore essential for mitigating the cytotoxic effects of ACR.

Our results revealed that H_2_S could be an ideal candidate for counteracting ACR-induced ferroptosis. It worked through multiple mechanisms. First, H_2_S enhanced cellular oxidative defenses. In contrary to the effects of ACR, H_2_S upregulated SLC7A11, increased intracellular GSH level and suppressed the activation of redox signaling pathway. In addition, H_2_S can also upregulate GPX4 expression (Zhang et al., 2024), a key enzyme that inhibits ferroptosis by converting lipid hydroperoxides into non-toxic lipid alcohols. Second, H_2_S restricted iron-mediated-catalytic ROS generation. H_2_S limited the availability of catalytic iron, essential for the Fenton reaction, thereby reducing ROS production and subsequent lipid peroxidation. Third, H_2_S directly eliminated lipid peroxidation products. Previous studies, including our own, have documented that H_2_S can directly scavenge ROS and lipid-derived aldehydes such as ACR and 4-hydroxynonenal (4-HNE) (Schreier et al., 2009; Mao et al., 2022; Mao et al., 2023a). In the current study, we also demonstrated that H_2_S dramatically suppressed ACR accumulation resulting from GPX4 inhibition. Collectively, our findings indicate that H_2_S offered multiple protective mechanisms against ferroptotic cell death induced by ACR.

Apart from ACR, H_2_S also inhibited GPX4 inhibitor RSL3-induced ferroptosis in Sertoli cells. Because ferroptosis induced by various insults shares similar molecular mechanisms, it is conceivable that H_2_S could act as a potent ferroptosis inhibitor irrespective of the specific insult. Indeed, there are several reports showing that H_2_S suppressed ferroptosis in several different cell types induced by different insults (Wang et al., 2020; Wang et al., 2022; Zhang et al., 2024). For examples, H_2_S inhibits specifically RSL3-mediated cell death in muscle and lung cells (Wang et al., 2020; Wang et al., 2022). Thus, our results add to the emerging evidence that H_2_S is a powerful inhibitor of ferroptosis across multiple cell types and different ferroptotic triggers. Our study provided the first evidence showing a critical involvement of H_2_S in protecting cells from ACR-induced ferroptosis in Sertoli cells. It may contribute to the defense against reproductive injuries. Notably, ferroptosis has been implicated in many diseases, including kidney dysfunction, neurodegeneration, and ischemia-reperfusion injury (Jiang et al., 2021). Thus, the findings in this study could also be used to mitigate ferroptosis and oxidative damage in these contexts.

It is worth mentioning that we have reported that H_2_S is a potent scavenger of ACR (Mao et al., 2022). The question naturally occurs as to whether the direct reaction between ACR and NaHS could influence the interpretation of the changes in the downstream molecular events. If the reaction between ACR and H_2_S caused a complete elimination of ACR, the downstream events should also be significantly affected. However, even so, it should not invalidate the overall conclusions because 1) NaHS alone significantly increases GSH and SLC7A11, suggesting that H_2_S can directly modulate ferroptosis pathways; 2) inhibiting endogenous H_2_S generation with BCA also significantly potentiated ACR-induced ferroptosis; 3) H_2_S also blunted RSL3-induced cell death, lipid peroxidation, and ACR accumulation (Figure 5), indicating that the protective effect of H_2_S extends beyond simply scavenging ACR. Collectively, this evidence supports the role of H_2_S in protecting against ferroptosis induced by ACR and other stimuli.

In this study, we chose to investigate Sertoli cells, as they are the main components of the BTB and provide the first line of defense against various insults (Kaur et al., 2014). Sertoli cells are known to be susceptible to both oxidative stress and inflammatory damage (Wong and Cheng, 2011; Dutta et al., 2021). Since Sertoli cells play a critical role in supporting germ cell growth and development through supplying nutrients, growth factors, and other biomolecules (Petersen and Söder, 2006), their dysfunction could greatly impact germ cell activity. Recently, ferroptosis has been shown to mediate Sertoli cell death induced by diverse factors. For example, ferroptosis drives oxygen-glucose deprivation/reoxygenation (OGD/R)-induced Sertoli cell death (Li et al., 2018). It also has a key role in mono-2-ethylhexyl ester (MEHP)-induced Sertoli cell injury (Wu et al., 2022). In addition, Hepatitis B virus decreases human Sertoli cell viability by promoting ferroptosis through TRIM37-mediated ubiquitination and loss of GPX4 activity (Pan et al., 2023). In this context, our study reveals a novel role for H_2_S as a master regulator of lipid peroxidation and ferroptosis in Sertoli cells. The finding may enhance our understanding of self-defensive mechanisms in Sertoli cells. As the results of the current study are based on *in vitro* cell models, further research is necessary to determine whether these findings can be replicated *in vivo*. This will be the primary focus of our future investigations.

It is worth mentioning that, as a ferroptosis inhibitor, H_2_S has several advantages. First, its inhibitory effect on ferroptosis is not insult-specific. It suppressed ferroptosis triggered by multiple inducers. Second, H_2_S blocks ferroptosis through diverse mechanisms. Third, as a gaseous transmitter, H_2_S readily permeates cell membranes (Riahi and Rowley, 2014), making it uniquely equipped to access lipid peroxides that form at membrane lesions.

In conclusion, our findings establish ferroptosis as a potentially important mechanism driving ACR-induced toxicity in Sertoli cells. Moreover, we have identified H_2_S as a potent protector against ACR-mediated ferroptotic cell death. Our study indicates that targeting the H_2_S pathway holds promise as a therapeutic strategy to alleviate oxidative damage and ferroptosis triggered by ACR and other insults as well.

## Data Availability

The raw data supporting the conclusions of this article will be made available by the authors, without undue reservation.
